# Electrically-triggered micro-explosion in a graphene/SiO_2_/Si structure

**DOI:** 10.1038/s41598-018-25776-z

**Published:** 2018-05-09

**Authors:** Siyang Liu, Myungji Kim, Hong Koo Kim

**Affiliations:** Department of Electrical and Computer Engineering and Petersen Institute of NanoScience and Engineering, 1238 Benedum, University of Pittsburgh, Pittsburgh, PA 15261 USA

## Abstract

Electrically-triggered micro-explosions in a metal-insulator-semiconductor (MIS) structure can fragment/atomize analytes placed on it, offering an interesting application potential for chip-scale implementation of atomic emission spectroscopy (AES). We have investigated the mechanisms of micro-explosions occurring in a graphene/SiO_2_/Si (GOS) structure under a high-field pulsed voltage drive. Micro-explosions are found to occur more readily in inversion bias than in accumulation bias. Explosion damages in inversion-biased GOS differ significantly between n-Si and p-Si substrate cases: a highly localized, circular, protruding cone-shape melt of Si for the n-Si GOS case, whereas shallow, irregular, laterally-propagating trenches in SiO_2_/Si for the p-Si GOS case. These differing damage morphologies are explained by different carrier-multiplication processes: in the n-Si case, impact ionization propagates from SiO_2_ to Si, causing highly-localized melt explosions of Si in the depletion region, whereas in the p-Si case, from SiO_2_ towards graphene electrode, resulting in laterally wide-spread micro-explosions. These findings are expected to help optimize the GOS-based atomizer structure for low voltage, small-volume analyte, high sensitivity chip-scale emission spectroscopy.

## Introduction

A graphene/oxide/semiconductor (GOS) structure forms a basic building block for electronic or optoelectronic device applications^1–5^. Similar to the case of a metal/oxide/semiconductor (MOS) capacitor a GOS structure can harbor two different types (inversion or accumulation) of two-dimensional electron system (2DES) at the oxide/Si interface, depending on the polarity of gate bias applied across the oxide layer. A switching control of this 2DES by oxide field is an essential function of MOS- or GOS-based field-effect devices. From the operation point of view, dielectric breakdown is an important reliability issue in microelectronics, and as a safeguard the maximum oxide field is usually designed to be limited far below breakdown field strength^6–10^.

We have investigated alternative application potential of GOS structure by driving it into a high-field breakdown regime. The resulting breakdown phenomenon is exploited as a means of fragmenting/atomizing analytes placed on top of the GOS structure, demonstrating atomic emission spectroscopy in an air-ambient room-temperature operation^11,12^. In brief, small-volume analytes (solid or liquid phase) were placed on graphene surface by physical vapor deposition, or spin/drop coating. Voltage pulses (~50 V) were applied to the sample (between top graphene electrode and bottom electrode on Si substrate) inducing local breakdown and explosions of GOS structure. This micro-explosion process resulted in fragmentation and atomization of analytes as well. The atomic emission emanating from fragmented analytes was then analyzed by a CCD-based spectrometer. In this GOS structure, graphene is designed to serve as an atomically-thin conducting electrode, on which analytes are placed. Besides excellent conductivity, graphene electrode offers chemical and physical stability, and does not produce any significant atomic emission (carbon lines) in the visible to near-IR range, therefore less prone to interference effects with analyte spectrum when compared with the metal electrode case (e.g., Ag)^11^. This electrically-triggered atomization on a micro-chip promises chip-scale miniaturization of an atomic emission spectroscopy (AES) setup, enabling a compact configuration and low-voltage/ambient operation with small volume analytes.

While our previous work had focused on demonstrating AES operation with analytes, in this article we have elucidated underlying mechanisms of micro-explosions occurring in a GOS structure without analytes under high-field voltage pulses of different polarities: inversion or accumulation bias on p-Si or n-Si substrate. Each configuration involves a different amount of inversion or accumulation charges with or without forming a depletion region in the capacitor structure. We analyzed the field distributions and carrier multiplications occurring in the GOS structure, and elucidated the governing mechanisms of dielectric breakdown, explosive melting and atomic emission. By performing microscopic analysis of explosion sites and comparing damage morphologies with simulation results we established a more detailed picture of breakdown/explosion process occurring in a GOS structure.

## Sample Preparation and Characterization

**Figure 1:**
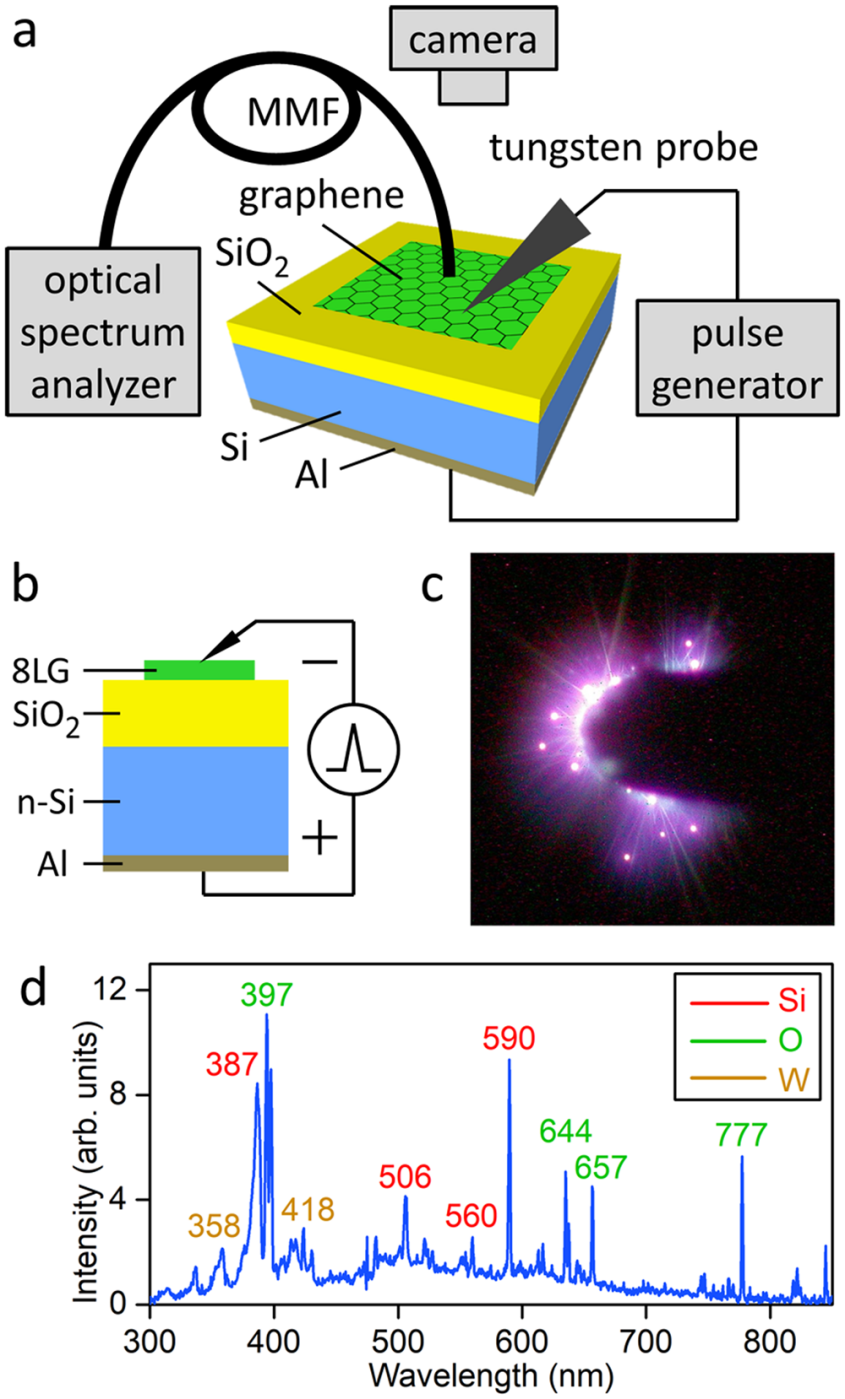
Electrically-triggered micro-explosion in a graphene/SiO_2_/Si (GOS) structure. (**a**) Schematic of an experimental setup for atomic emission spectroscopy. (**b**) Schematic of a GOS capacitor structure under an inversion-bias pulsed drive: 8-layer graphene (8LG)/10-nm SiO_2_/n-Si. (**c**) Optical micrograph taken during micro-explosions. (**d**) Atomic emission spectrum measured from an n-Si GOS sample.

A 10-nm-thick SiO_2_ layer was grown on (100)-Si wafer (p-type or n-type; 10 Ω-cm resistivity) by thermal oxidation. An Al (100-nm thick) Ohmic contact was prepared on the bottom side via thermal evaporation deposition. An 8-layer graphene (8LG: CVD grown on a Cu foil, purchased from ACS Material) was transferred onto the SiO_2_ layer as a top (gate) electrode in the following procedure^13^. A PMMA layer (MicroChem 950 PMMA A7) was spun-coated on the graphene-covered Cu foil. The Cu foil was etched away in ferric chloride solution (Transene Cu Etchant CE-100). The PMMA/graphene stack was transferred to SiO_2_/Si substrate. The PMMA was removed in acetone, leaving graphene only transferred to substrate. Electrical pulses (50-V amplitude; 10-μs pulse width; 1-s interval) were applied to the GOS structure by a pulse generator (HP 214B) (Fig. 1a). A tungsten probe was used to access the top electrode (8LG). Figure 1c is an optical micrograph taken during a pulsed operation showing emission of light. Figure 1d shows atomic emission lines from an 8LG/10-nm-SiO_2_/n-Si GOS sample captured by a CCD-based optical spectrum analyzer.

## Field Distribution

The field distributions in the GOS structure were analyzed (see Supplementary Information, Section 1. Calculation of electric field distributions in GOS structure). The gate voltage (V_g_) applied to a GOS structure can be expressed as a sum of flat band voltage (V_FB_), surface potential (φ_S_) and oxide voltage drop (V_ox_). Here flat band voltage (V_FB_) is equal to graphene work function (ϕ_gr_) minus silicon work function (ϕ_Si_). The voltage drop across oxide layer (V_ox_) is related to the space charge density (Q_S_) and oxide capacitance (C_ox_) as follows: V_ox_ = Q_S_/C_ox_. The space charge density in Si can be obtained by solving Poisson equation [see Supplementary Information reference S1]. Graphene work function is the sum of intrinsic graphene work function (4.56 eV) and its Fermi level shift (ΔE) under electric field.

In this study both monolayer graphene and 8-layer graphene (CVD grown on Cu foil; purchased from ACS Material) were investigated as a top electrode, placed on SiO_2_/Si substrate by a transfer method. In terms of endurance over multiple voltage pulses, thicker graphene (8-layer graphene) offers better mechanical stability than monolayer graphene and allows more explosions, which results in stronger emission spectra (cumulative). Therefore, in experimental work, we focused on 8-layer graphene case. In analytical calculation, we considered both cases and compared the calculation results. The bias-voltage dependence of Fermi level in the graphene electrode layer was taken into account in this calculation^14^: see Supplementary Information Section 1. For the monolayer graphene case the following relationship is assumed:

1 $${\rm{\Delta }}E=\pm \,\hslash |{\nu }_{F}|\sqrt{\pi {n}_{s}}$$

where n_s_ denotes carrier concentration in graphene and is related to space charge density in Si side (Q_S_) as follows, n_s_ = Q_S_/q. For the 8-layer graphene case we assumed the following relationship (bilayer graphene model)^15^:

2 $${\rm{\Delta }}E=\pm \,{\hslash }^{2}\pi {n}_{s}/2{m}^{\ast }.$$

It has been well known that the voltage dependence of graphene’s Fermi level becomes negligible when the number of graphene layers is greater than 2^15^.

By solving the above equations simultaneously we calculated electric fields in the oxide layer (E_ox_), at the Si/SiO_2_ interface (Si side) (E_int_), maximum depletion field (E_dpl_), depletion width (W), and inversion layer thickness (t_inv_) at 10 V, 30 V or 50 V bias. Unlike the case of monolayer graphene, whose Fermi level may significantly shift depending on bias^14,16^, the 8-layer graphene is found to show a negligible amount of Fermi level shift in this bias range (up to 50 V). At 50 V bias, for example, calculation shows that the Fermi level of monolayer graphene shifts by 1.3–1.8 V (see Supplementary Figures 1a,b). In the bilayer graphene case, the Fermi level shift remains <0.1 V at 50 V bias. For 8-layer graphene case, the Fermi level shift is expected to be even smaller.

**Figure 2:**
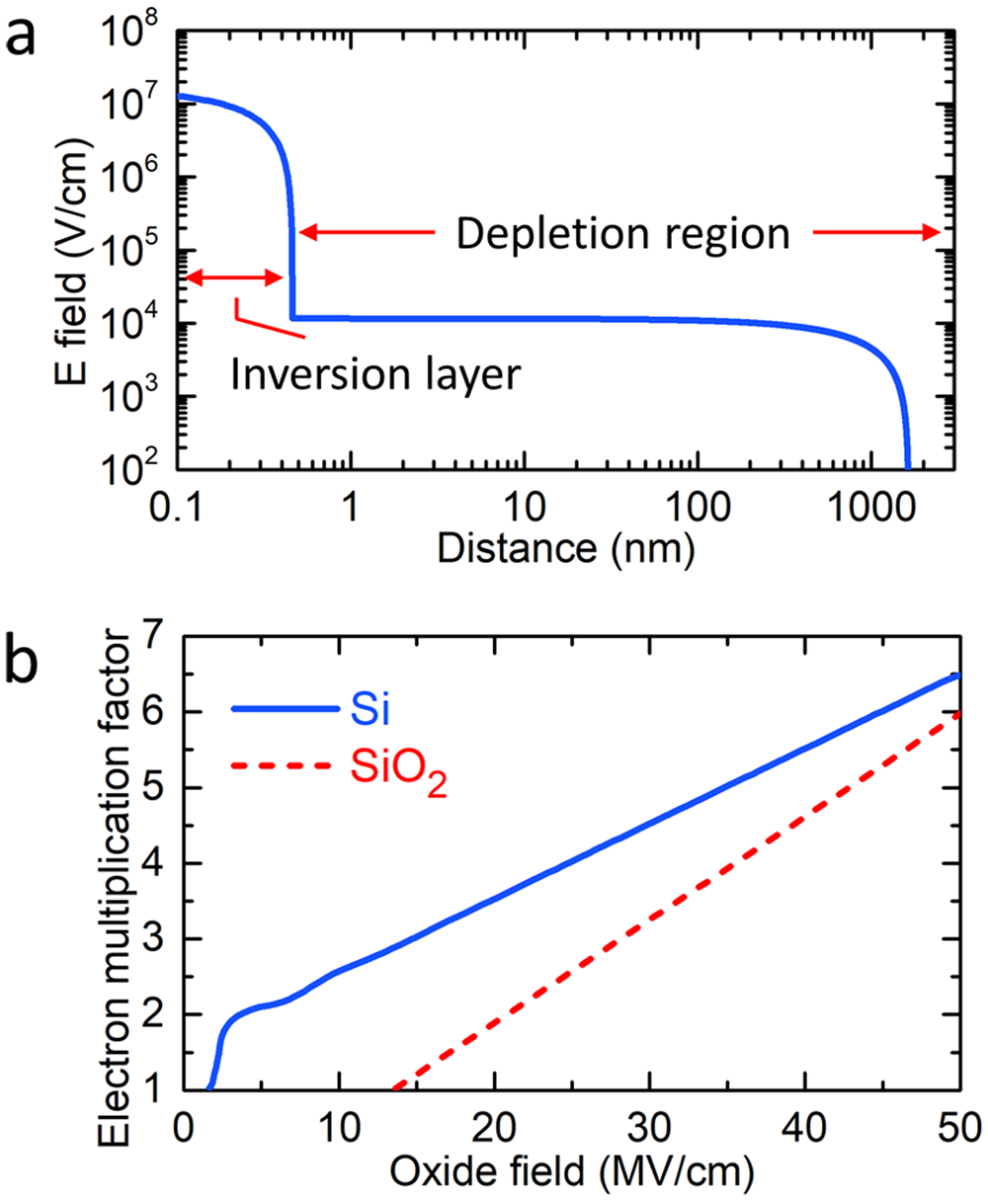
Field distribution and carrier multiplications in a GOS structure. (**a**) Electric field distribution in 8LG/SiO_2_(10 nm)/n-Si under 50-V inversion bias: calculated before a breakdown process embarks. (**b**) Electron multiplication factors calculated as a function of oxide field: in SiO_2_ layer (dashed) and in Si depletion region (solid).

Under strong inversion bias both n-Si and p-Si GOS samples demonstrate similar field distributions: the depletion region width saturates at 1.0 µm or 1.6 µm for p-Si or n-Si case, respectively, that is, most of the applied voltage drops across the 10-nm SiO_2_ layer, resulting in a field strength of ~48 MV/cm in SiO_2_ and ~10 kV/cm in Si depletion region at 50 V bias. Figure 2a shows a log-log scale plot of field distribution in the 8LG/SiO_2_(10 nm)/n-Si GOS sample at 50 V inversion bias. Note that the strong field (~16 MV/cm) in Si side is narrowly confined to an inversion layer whose thickness is ~1 nm. The field then sharply drops to ~10 kV/cm level inside the depletion region (at the interface of inversion layer and depletion region). Over the depletion region width (~1.6 µm) the field monotonically decreases to zero at the edge of depletion region. In brief, the bias voltage (50 V) applied across the 8LG/SiO_2_(10 nm)/n-Si sample distributes with the following division: 48.8 V across SiO_2_ and 1.2 V across Si depletion (including the voltage drop across the inversion layer).

## Carrier Multiplications

When driven into such a high field regime, the strong field in the oxide layer can trigger impact ionization. Impact ionization is the process that an energetic charge carrier (electron or hole) generates new electron-hole pairs by losing its energy through a collisional process^17–21^. Ionization rate is defined as the number of electron-hole pairs generated per carrier per unit distance traveled, and, in general, is a strong function of electron energy. Being a band-to-band process requiring relatively large energy (>9 eV, SiO_2_ bandgap), impact ionization in SiO_2_ is known to be insignificant in a low-field regime^17,21^. At relatively high fields (>~10 MV/cm) and sufficient oxide thickness (>~10 nm), however, electrons in graphene can tunnel into SiO_2_ via Fowler-Nordheim (FN)-tunneling process and subsequently can gain a significant amount of energy from the oxide field: the mean free path in SiO_2_ is known to be ~4 nm^22^; in the high-field regime calculated above (e.g., oxide field of ~48 MV/cm at 50 V bias for n-Si case) tunnel-injected electrons in SiO_2_ would gain, on average, ~19 eV kinetic energy from the oxide field during this scattering-free transport^17^. In this high-field regime, high-energy tails are known to rapidly develop with increasing field strength, extending beyond the average gain estimated above^17,18^. This high energy range available from these hot electrons far surpasses SiO_2_ bandgap, and therefore induces impact ionizations in the oxide layer.

Carrier multiplication factor (*m*) is defined as the ratio of output and input current densities of one kind of carriers (electrons or holes)^23–25^, and can be obtained by integrating ionization rate over distance. Figure 2b (dashed) shows the electron multiplication factor in SiO_2_ calculated as a function of oxide field (*E*_ox_) for oxide thickness (*t*_ox_) of 10 nm, referring to the following formula, which takes into account the high-energy tail effects in a high-field regime^17,18^:

3 $$m=1+P(\frac{{E}_{ox}}{{F}_{th}}-1)$$

4 $$P={P}_{1}\frac{1}{{t}_{ox}-{t}_{d1}}$$

5 $${F}_{th}={F}_{th}^{\infty }(1+\frac{{t}_{1}}{{t}_{ox}-{t}_{d2}})$$

where *P*_1_ = 5.5 *nm*, *t*_*d*1_ = 7 *nm*, ${F}_{th}^{\infty }=3.8\,MV/cm,{t}_{1}=21.6\,nm,{t}_{d2}=1.5\,nm$.

A multiplication factor of 5.5 is expected to be obtainable at 50 V bias: see Fig. 2b (dashed).

In a strongly depletion-biased GOS, a further carrier multiplication is expected to occur in Si. During the transport through a SiO_2_ layer both (phonon-) scattered and (impact-ionization-) generated electrons will gain momentum (kinetic energy) from the field, becoming hot again. Upon entering Si side (and until next scattering/collision event) the injected electrons will also gain extra potential energy with the amount corresponding to the conduction band offset (3.2 eV) at SiO_2_/Si, which subsequently converts to kinetic energy. These highly kinetic electrons can induce more impact ionizations in Si depletion region. The total energy (*E*_e_) of an electron impinging upon Si can be calculated from the following formula^26,27^:

6 $${E}_{e}=q{E}_{ox}\lambda (1-{e}^{\frac{{s}_{ox}-{t}_{ox}}{\lambda }})+q{\varphi }_{b}$$

where *λ* denotes mean free path of electron in oxide; *s*_ox_ is the tunneling distance; *ϕ*_b_ is the barrier height (conduction band offset) at the injection side. This model basically assumes the following energy balance relationship during transport inside SiO_2_: Fowler-Nordheim-tunneled electrons (from graphene to SiO_2_) will go through two competing processes, that is, gaining kinetic energy from oxide field while losing energy through collision/scattering after every mean-free-path propagation.

The carrier multiplication factor (*m*) in Si can be expressed as^26–28^

7 $$m=1+{\sum }_{n=0}^{\infty }n{p}_{n}({E}_{e})$$

where *p*_n_ (*E*_e_) is the probability that an electron with energy *E*_e_ ultimately creates *n* pairs of electron-hole. Combing the two results (Eqs 6 and 7: incident electron energy *E*_e_ versus oxide field *E*_ox_; carrier multiplication *m* versus incident electron energy *E*_e_), the multiplication factor in Si is calculated as a function of oxide field: see Fig. 2b (solid). According to Eq. 6, at 50 V bias the average energy of electrons exiting the 10-nm oxide can reach 20-eV level. These highly-kinetic electrons, when impinging upon Si side, produce a carrier multiplication of 6: see Fig. 2b (solid). Being cascaded, therefore, multiplicative, the overall multiplication factor is estimated to be greater than 30, that is, 5.5 (in SiO_2_) times 6 (in Si). The escalating nature of this two-stage multiplication process in an inversion-biased n-Si GOS implies that the Si depletion region would be easily flooded with a significantly larger amount of current flow than that in SiO_2_.

By contrast the accumulation bias case presents a different picture (that is, absence of depletion region in Si side) and differing characteristics compared with the inversion bias case: our experimental work demonstrates that an inversion bias operation more readily (i.e., at lower bias) produces stronger atomic emission than the accumulation bias for both p-Si and n-Si GOS cases, implying easier breakdown at inversion bias. This differing breakdown characteristic can be explained as follows. In the case of accumulation-biased p-Si GOS, for example, impact ionization initially generates electron-hole pairs in SiO_2_ under strong oxide field, similar to the inversion biased n-Si case discussed above: note that most of the applied voltage at accumulation bias drops across the oxide layer, resulting in the same field strength, ~50 MV/cm, as in the inversion bias case; the generated electrons then travel down and are injected into Si, in the same direction as in the inversion biased n-Si; inside Si, however, accumulation holes (majority carriers in p-Si) are awaiting and readily recombine with incoming electrons. Overall, the absence of a depletion region (i.e., presence of accumulation holes) adjacent to the oxide layer is found to make a critical difference, disabling carrier multiplication inside accumulation-biased p-Si.

It is noteworthy that the band offset at SiO_2_/Si interface significantly differs for conduction and valence bands: 3.15 eV for conduction band and 4.17 eV for valence band. Besides the depletion region effect discussed above, this band offset difference might have played a certain role in determining a preferred injection mode. In a p-Si GOS structure, for example, inversion electrons in Si are injected into SiO_2_ under inversion bias, whereas majority holes in Si are injected into SiO_2_ under accumulation bias. The large energy-barrier difference would favor electron injection into SiO_2_ over hole injection, therefore an inversion bias would be a preferred mode for the p-Si case in terms of initiating impact ionization in SiO_2_. In the n-Si GOS case, however, the situation is different: inversion holes in Si are injected into SiO_2_ under inversion bias, whereas majority electrons in Si are injected into SiO_2_ under accumulation bias. This time the band offset difference would prefer accumulation bias (for electron injection) over inversion bias (for hole injection) from the carrier injection perspective, therefore for initiating impact ionization in SiO_2_. Quite interestingly our experiment with n-Si samples demonstrate that inversion bias allows explosions more readily/easily than accumulation bias case, although the latter would perform better in initiating impact ionization in SiO_2_. Overall this comparison confirms that presence of a depletion region is an essential requirement for micro-explosions: impact ionization in SiO_2_ itself without backup from a depletion region does not produce large multiplication factors. Considering this intrinsic difference, that is, the difficulty in inducing breakdown/explosion in accumulation bias, this study focuses on the inversion bias cases of both n-Si and p-Si GOS structures and compares their underlying mechanisms.

## Explosion Damages

**Figure 3:**
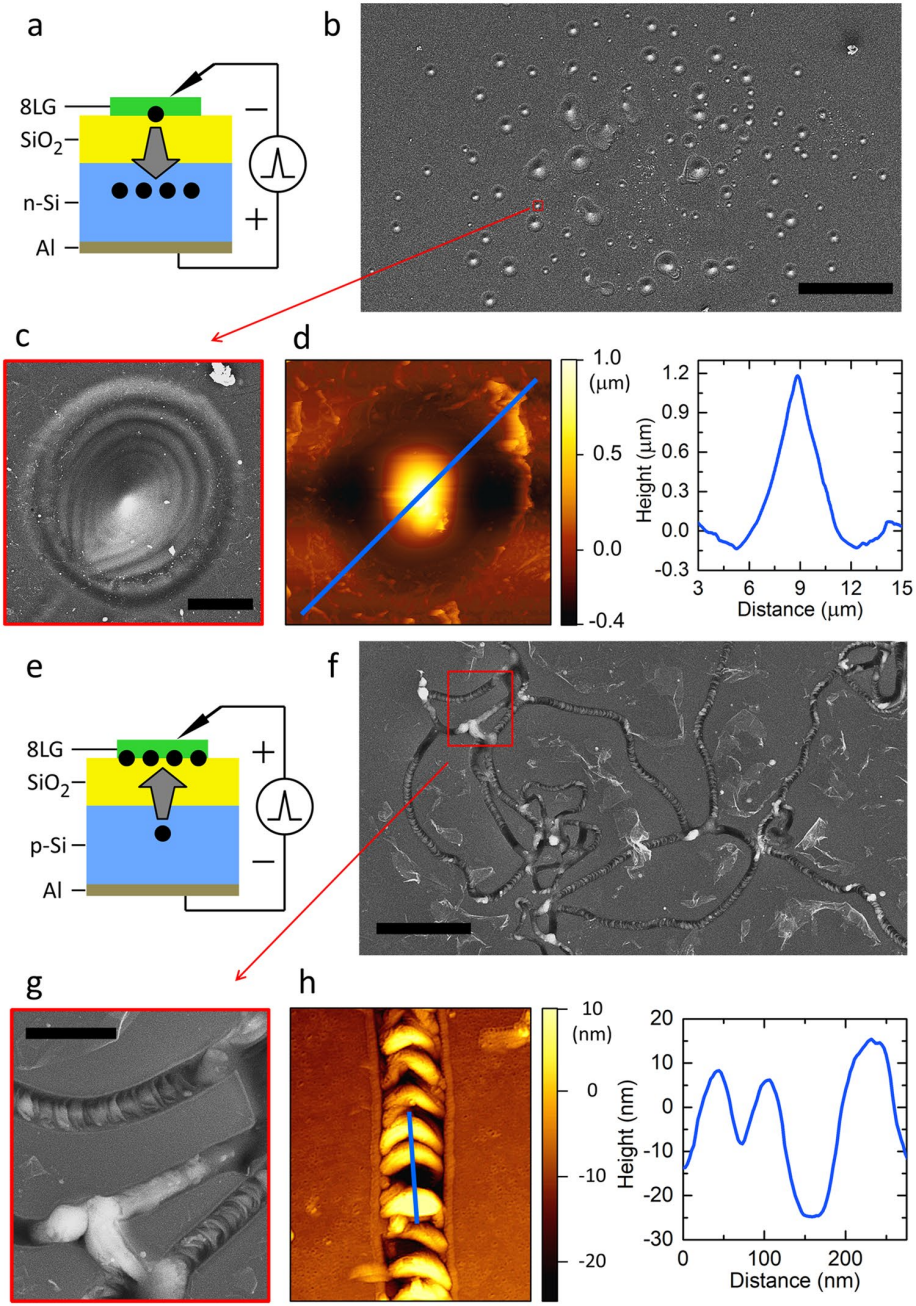
Micro-explosion damages: n-Si GOS (**a**–**d**) and p-Si GOS (**e**–**h**). (a) Schematic of sample structure and bias: inversion bias to n-Si GOS. The arrow indicates the direction of electron flow. (b) SEM image of micro-explosion damaged sites. Scale bar, 100 µm. (c) Magnified view of a damaged site. Scale bar, 5 µm. (d) AFM scan image (left) and profile (right) of a damaged site. Note the circular, protruding cone-shape profile of Si melt. (e) Schematic of sample structure and bias: inversion bias to p-Si GOS. The arrow indicates the direction of electron flow. (f) SEM image of micro-explosion damaged sites. Scale bar, 2 µm. Note the random network of narrow trenches. (g) Magnified view of a damaged site. Scale bar, 500 nm. (h) AFM scan image (left) and profile (right). Note the crescent profile of melt undulations within a trench.

Figure 3a–d shows scanning electron microscopy (SEM) and atomic force microscopy (AFM) images of n-Si GOS samples tested with inversion bias pulses (i.e., negative voltage to graphene electrode). Highly localized, circular, protruding eruption damages are observed. The damage extent is measured to be ~1.2-µm-high as shown in Fig. 3d. The base diameter is measured to be 1–30 µm. This large feature size suggests a major explosion occurred at each eruption site. It is also noteworthy that these eruption sites are well isolated and circularly symmetric with a well-defined cone-shape profile (i.e., protruding at the center). It is interesting to note that the damage depth (0.2–1.0 µm) closely matches the depletion region width (~1 µm) in Si side. This suggests a large amount of current flew inside/through the Si depletion region, causing Joule heating and melting of Si. The ripples (concentric rings: Fig. 3c) in the edge area of each cone indicate the wave motion of molten material induced by shock loading^29^, and support the melt-explosion hypothesis discussed above.

Totally different damage morphologies are observed in the case of p-Si GOS under inversion bias (i.e., positive voltage to graphene electrode) as shown in Fig. 3e–h. A network of randomly-distributed trenches is observed on the sample surface. The trenches have a meandering and branched pattern. Most part of the network is well connected, indicating that the trenches formed in a laterally propagating mode during explosions^30,31^. The trenches have an average width of ~200 nm and a depth of 20 nm. This trench depth indicates that the damage extends to ~10 nm depth of Si surface. The graphene electrode around eruption sites is fragmented/detached into flakes by explosions as shown in Fig. 3f. The relatively shallow trench depths observed in p-Si GOS samples indicate that explosions occurred primarily in/around the oxide layer and the damage extent is confined to near SiO_2_/Si interface, much smaller than the inversion biased n-Si GOS case.

## Mechanisms of Micro-Explosions

**Figure 4:**
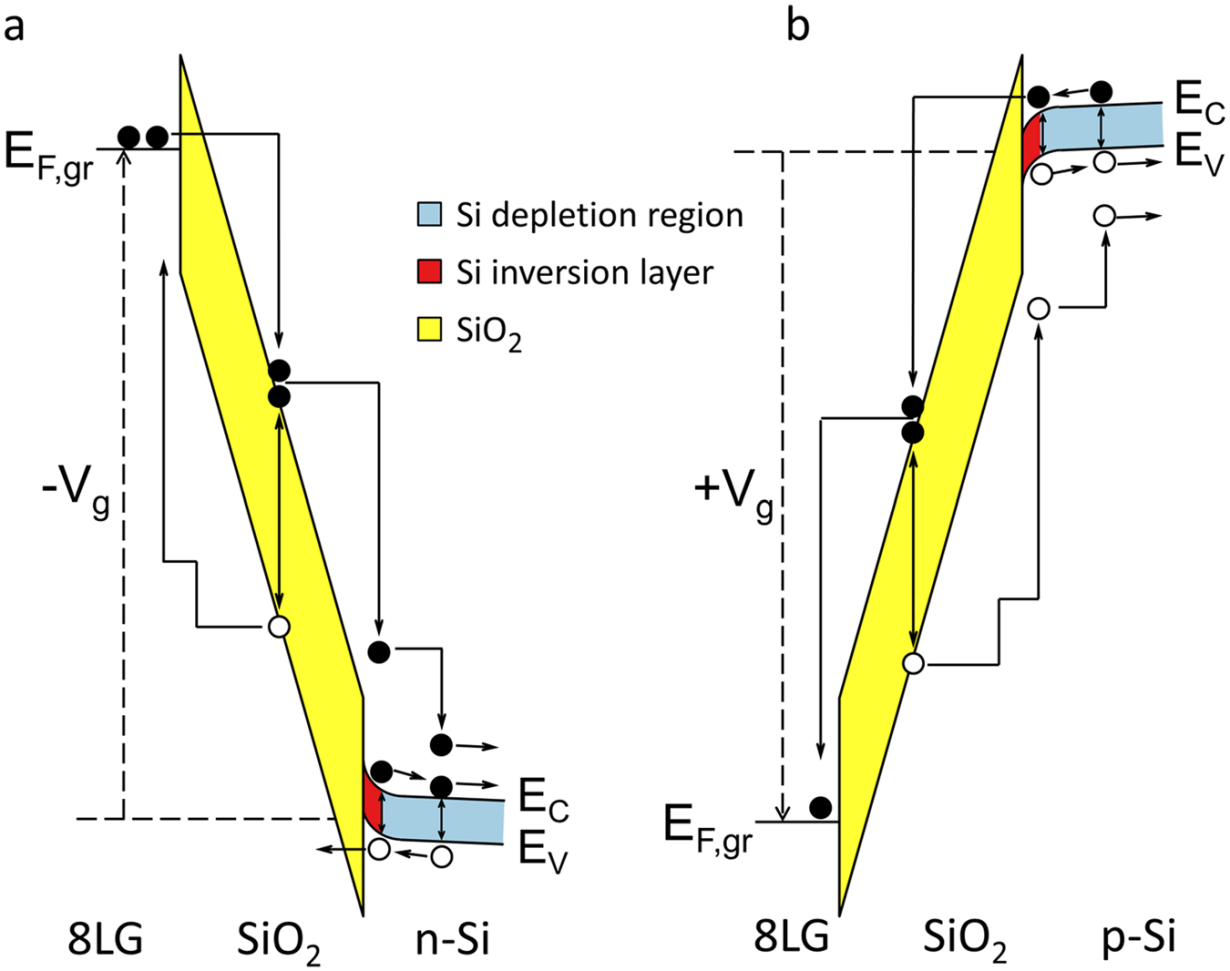
Carrier multiplications in GOS structure. (**a**) n-Si GOS under inversion bias. Note the cascade of impact ionizations occurring in SiO_2_ and Si depletion region. The carrier multiplication intensifies in Si side. (**b**) p-Si GOS under inversion bias. The carrier multiplication intensifies towards graphene side. Note also the positive feedback effect induced by impact-ionization-generated holes in SiO_2_.

Figure 4 illustrates schematics of carrier multiplication processes occurring in the n-Si and p-Si GOS structures under inversion bias. In the n-Si case (Fig. 4a), electrons are injected into SiO_2_ from the graphene side via Fowler-Nordheim tunneling; the injected electrons induce impact ionization, generating electron-hole pairs in SiO_2_; the generated electrons drift down towards Si side: when entering Si, the electrons gain extra energy (3.2 eV) and further induce impact ionization in Si depletion. Being multiplicative, the carrier generation process then intensifies inside Si. The holes generated there will be separated by depletion field and drift up toward SiO_2_. The depletion region (~1 µm width) becomes easily flooded with incoming electrons and newly generated electrons/holes there. The large current flow in the depletion region will result in Joule heating, which would cause explosive melting of Si and SiO_2_.

It is interesting to note that a center-protruding cone-shape profile is persistently observed from those melt-explosion sites of inversion-biased n-Si GOS samples. This protruding cone-shape resembles ‘Taylor cones’, which have been widely reported in literature^32–35^. According to Taylor^32^ a liquid surface under strong electric field will form into a cone-shape, where the electrostatic stress acting on liquid surface is balanced by the surface tension of the melt. The Taylor-cone formation observed in the present study is explained by a similar mechanism, that is, Coulombic interaction of Si melt with bias field: first, highly kinetic incident electrons bombard and impact-ionize Si; Si melts by Joule heating under a large current flow; positively-charged Si ions in the melt are pulled upward to the graphene side by the inversion bias field (i.e., negative voltage to graphene cathode), shaping the melt into a protruding cone-shape profile (Fig. 3d). It is worth mentioning that the opposite profile (i.e., a protrusion of cathode side surface) would be expected in the case of electro-migration, which normally occurs in a solid phase environment and to the direction of electron flux^9^. The discrete nature of damaged spots in the n-Si case can be ascribed to the relatively poor adhesion/integrity at the graphene/SiO_2_ interface. When an explosion occurs at a particular site, the graphene electrode is easily fractured and detached around that area. Note that in this inversion-biased n-Si GOS case a carrier multiplication process is initiated by electron injection from the graphene side (Fig. 4a). Once the local area of graphene electrode becomes detached by an explosion, there cannot be further explosion in the same/nearby area because of the difficulty in electron injection into SiO_2_ from the detached graphene cathode.

In the p-Si case (Fig. 4b), inversion electrons in Si are injected into SiO_2_ via Fowler-Nordheim tunneling process: note the opposite direction of electron flow compared to the inversion-biased n-Si GOS case. In the high-field regime tested in this work, impact ionization occurs in SiO_2_ generating electron-hole pairs. The generated electrons will drift towards graphene side inducing further impact ionizations: the carrier multiplication process becomes stronger (i.e., more frequently occurring) toward the graphene side of SiO_2_ like an avalanche process. The holes generated in SiO_2_ will also drift to Si side. When entering Si, the holes become hot, suddenly gaining extra potential energy that corresponds to the valence band offset (4.2 eV). These highly kinetic holes can generate electron-hole pairs in the depletion region. While the generated holes travel down towards Si substrate, the extra electrons will be injected back to the SiO_2_ layer; the injected electrons will join impact-ionization-generated electrons in SiO_2_, contributing to the carrier multiplication process there (inside SiO_2_). This positive feedback effect, being enabled by the presence of a depletion region in Si, is considered to be critical to carrier multiplication in the inversion-biased p-Si GOS case. The relatively shallow damage depths (i.e., ~20 nm $\ll$ depletion region width) observed with p-Si samples support this breakdown mechanism: the electron-initiated carrier multiplication process in SiO_2_ becomes stronger towards the graphene side with a growing electron flux, flooding the oxide layer with a larger current flow compared with that in Si side.

The explosion damages of inversion-biased p-Si GOS samples reveal laterally-extended (contiguously propagating) defect profiles. Note that in the p-Si case the carrier multiplication in SiO_2_ is initiated by electron injection from Si side (Fig. 4b). The adhesion of a thermally-grown SiO_2_/Si interface is known to be excellent, and the integrity of adjacent area is unlikely to be affected by the shallow/local micro-explosions, allowing continual injection of electrons into SiO_2_. The oxide breakdown process can laterally propagate into adjacent weak spots: breakdown damages then become connected forming a meandering pattern as shown in Fig. 3f. The corrugation profile observed in the trench bottom suggests that local melt-explosions occurred and continuously migrated to adjacent sites leaving behind a characteristic wavy (crescent) pattern after each micro-explosion. The damage propagation direction can be inferred from this corrugation pattern: e.g., a downward propagation in the case of Fig. 3h.

## Lateral Propagation of Micro-Explosions

**Figure 5:**
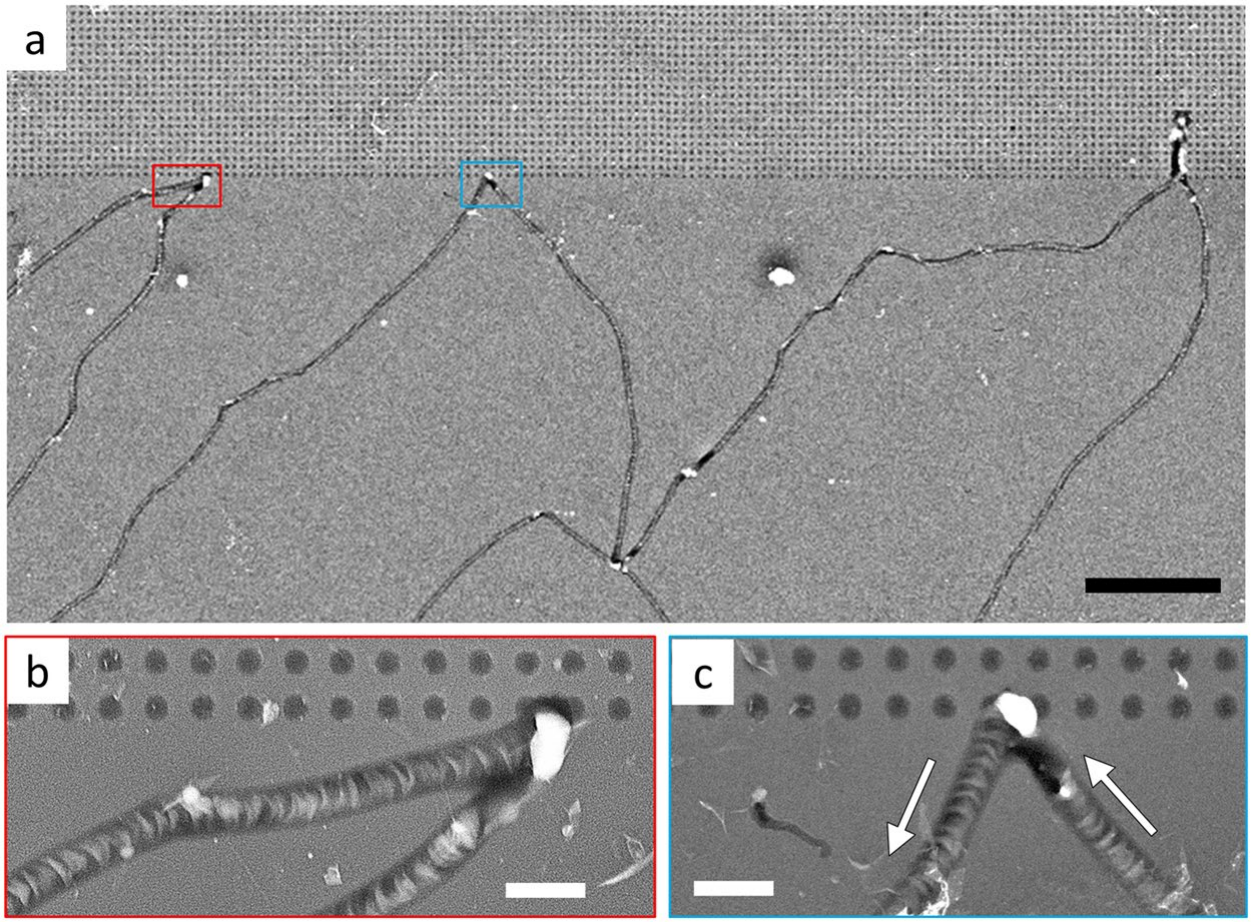
Lateral propagation of micro-explosions in p-Si GOS with a nano-hole array. (**a**) SEM image of micro-explosion-damaged area. Three trails (narrow trenches) propagated toward the nano-hole array patterned area (upper part) and bounced back. Scale bar, 5 µm. (**b**) Magnified view of the left-most trail. Scale bar, 500 nm. (**c**) Magnified view of the center trail. Damage propagation direction is marked with arrows. Scale bar, 500 nm.

The underlying mechanisms of laterally-propagating micro-explosions observed with inversion-biased p-Si samples were further investigated by employing a nano-hole-array patterned GOS structure (Fig. 5). The nano-hole GOS samples (p-Si) were fabricated in the following steps: first, a nano-hole array (100-nm diameter, 500-nm spacing and 85-nm depth) structure was formed on 10-nm-SiO_2_/Si substrate by employing e-beam lithography and reactive ion etching; then a CVD-grown 8-layer graphene (8LG) was transferred/suspended on the nano-hole-etched SiO_2_/Si substrate. Voltage pulses (50-V amplitude) were applied to the nano-hole samples. Laterally-propagating explosion damages (i.e., a trench pattern of closely-spaced eruption sites) were observed. Figure 5a shows three trails that bounced back when hit the nano-hole patterned area (upper part). In the case of middle one (Fig. 5c), the fringe (crescent profile) patterns that each local micro-explosion left behind indicate that micro-explosions have propagated to the north-west and bounced back to the south-west when they encountered the nano-hole array area. This traveling and bouncing behavior reveals a self-avoiding characteristic (i.e., avoiding a void area) of dielectric breakdown^31^ and can be understood as follows.

Carrier multiplication (therefore micro-explosion) would not occur in a pre-existing nano-hole area (i.e., the region with void channels formed in the oxide layer), because injected electrons will travel through the void without experiencing scattering/collision, therefore without inducing impact ionization. Also, it should be reminded that the carrier multiplication process in p-Si based GOS (without void channels) involves a positive feedback effect (i.e., an injection of electron-initiated impact-ionization-generated holes into Si induces a further injection of electrons into SiO_2_) as discussed above. By contrast, in the nano-hole array GOS case, holes cannot be injected into the void and therefore there would be no positive feedback effect, making explosion a difficult event to occur there.

The self-avoiding (i.e., avoiding void regions) and laterally propagating nature of micro-explosions observed with GOS samples without nano-holes is explained by a similar mechanism: when a void channel develops in an oxide layer, the carrier multiplication process discontinues in that area. Inversion electrons will then look for fresh sites nearby and make new explosions there. This explosion process will continue with random migration, forming a meandering pattern. Formation of a branch-like network pattern (see Figs 3f and 5a, bottom area) is explained by this self-avoiding behavior of micro-explosions: explosion stops to propagate and bounces back when it hits pre-existing void channels (trenches), similar to the case of bouncing off at the edge of a nano-hole array discussed above.

## Comparison with Low-Field Breakdown Mechanisms

We have investigated the physical mechanisms of micro-melt-explosions occurring in a GOS structure under a high-field pulsed drive. We now compare the results with low-field breakdown mechanisms. Breakdown at low fields ($\ll$breakdown field strength) is known to be initiated by hot-carrier injection into oxide: defects and trap states are then generated inside and/or near the interface of oxide by tunnel-injected electrons, having the effects of increasing local field and decreasing the barrier height for electron injection (i.e., a positive feedback effect)^36^. Here it should be noted that defect formation, having relatively low threshold energy (<9 eV), is considered to be a dominant process in a low field regime, as opposed to the band-to-band impact ionization in a high field regime. As defects accumulate, they form localized conduction paths through a percolation process. The defect channels thus-formed are known to have ~1 nm diameter, and a large current flowing through densely populated percolation channels results in Joule heating and thermal damage of dielectric^9^.

Here the following comparisons can be made between defect channels and nanoscale void channels. First, defect-generated percolation channels differ from the lithographically-defined void channels discussed above, in terms of their channel dimensions and carrier transport mechanisms. When the diameter of a void channel becomes relatively large (i.e., >~10 nm) the carrier transport process would be scattering-free and ballistic. As a result, impact ionization and carrier multiplication in a void channel are difficult to occur. Hole injection and transport are also prohibited inside a void channel. In terms of breakdown process, further evolution of percolation channels would stop at an intermediate point due to a balancing effect: as the channel diameter grows, the carrier transport becomes ballistic, resulting in less scattering and thermal damage and eventually leading to no further increase of channel diameter. In other words, in the high-field operation case, percolation channels would not grow into larger diameter void channels. Instead, the initial, slow defect-generation process will be quickly taken over by a much more rapid process of impact ionization and carrier multiplication occurring in SiO_2_ and Si as discussed above, which induces Joule heating therefore melt explosion of Si.

## Taylor Cones and Atomic Emission

**Figure 6:**
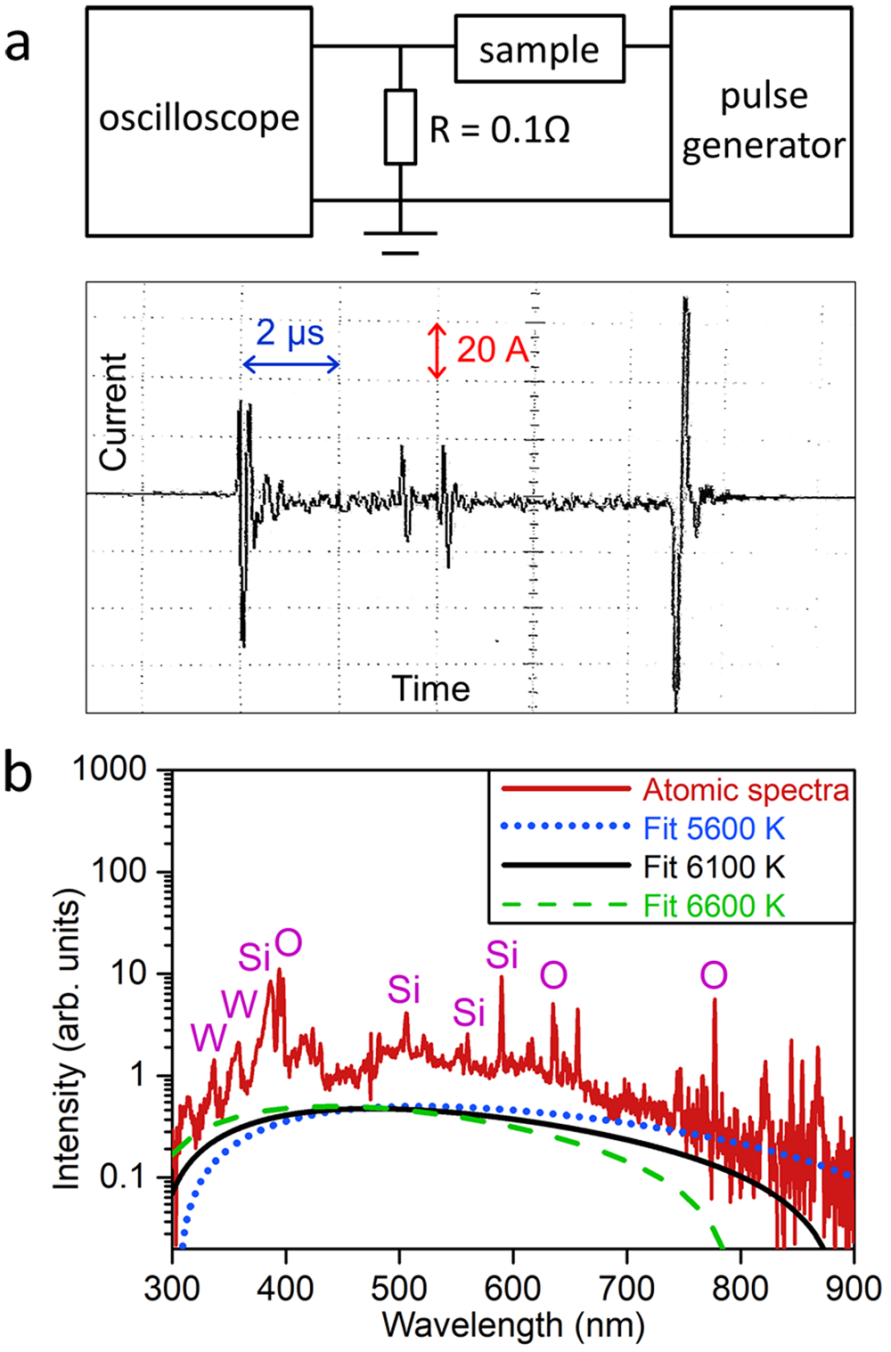
Electrically-triggered micro-explosions and atomic emission from GOS. (**a**) Schematic of current measurement during micro-explosions (top); measured current (bottom) from a n-Si GOS under inversion bias. (**b**) Optical emission spectrum measured during micro-explosions. Note the sharp atomic lines (labeled: S, O and W) and a blackbody radiation (broad continuous spectrum). The blackbody temperature is estimated to be ~6000 K (black solid curve fit).

In order to substantiate these findings we characterized the current flow through a GOS sample during a pulsed operation (Fig. 6a). 50-V, 10-µs pulses were applied to an n-Si GOS. 30-to-40 current spikes were observed during 10-µs pulse width with peak amplitude in the range of 1 to 20 A: each individual spike corresponds to a local micro-explosion occurring in the sample. The emission photo and SEM image (Figs 1b and 3b) show that each explosion site well matches bright emission spots, from which strong atomic emission emanates: note that the ray-like emission pattern from explosion sites indicates flying/radiating atoms generated by melt explosion (Fig. 1b). The amount of energy consumed for a micro-explosion, which went into Joule heating, can be estimated from the measured current spike information (peak current and pulse width): e.g., taking the largest spike with 20-A amplitude (Fig. 6a, bottom panel) the amount of Joule heating is estimated to be ~100 µJ (i.e., 50 V × 20 A × 100 ns). This amount of energy input is compared to be greater than the amount of energy (~15 µJ) needed for vaporization of Si at micro-explosion site: see Supplementary Information Section 2. Calculation of thermal energy needed for vaporization of Si. This comparison supports the melt-explosion mechanism discussed above.

Figure 6b shows a log-scale plot of emission spectrum from an inversion-biased n-Si GOS sample, captured during multiple pulses. A broad spectrum of UV, visible to NIR emission is observed while discrete atomic lines are registered with stronger intensity. This broadly continuous spectrum is attributed to a blackbody radiation, and the corresponding blackbody temperature is estimated to be ~6000 K (Fig. 6b, see the solid curve fit). This blackbody temperature surpasses the boiling point of Si (3538 K), and supports the mechanism that the micro-explosions observed in inversion-biased GOS samples are basically melt-explosion of Si triggered by impact ionization in SiO_2_. According to the spectral analysis of these GOS samples^11^ most of the Si emission lines observed are from singly- or doubly-charged Si ions: e.g., 387 nm (Si II), 506 nm (Si II), and 590 nm (Si III). The impact ionization energy of Si^+^ is 16 eV^37^, and the observation of atomic emission from charged Si atoms is consistent with the field analysis discussed above showing the availability of highly kinetic (~20 eV) incident electrons. In conjunction with the explosion topography (i.e., center-protruding) this information on the origin of atomic emission suggests the following mechanism of atomization: Si melt evaporates into atoms at the tips of Taylor cones via a field desorption process, which might be followed by inelastic collisions with atoms and electrons and/or field ionization^33–35^. Here the field desorption process, being enabled by field enhancement at the apexes of Taylor cones^33^, is considered to be a critical step in atomizing Si melt.

It is also noteworthy that confined micro-explosion has also been reported in literature by employing a tightly focused fs-laser pulse inside transparent material^38^. High energy density (up to several MJ per cm^3^) generates TPa-range pressure forming super-dense material phases and ion separation in hot non-equilibrium plasma of confined micro-explosion. While the laser-induced micro-explosion method is viewed to be more controllable in terms of forming nanoscale protrusions at predetermined places, the electrically-triggered micro-explosion reported here offers alternative advantage and application: a simple setup and relatively low voltage operation for atomizing small volume analytes. This advantageous feature is expected to be important in developing chip-scale instrumentation of atomic emission spectroscopy.

In summary, we have investigated the physical mechanisms of micro-explosions occurring in a graphene/SiO_2_/Si (GOS) structure under an inversion-bias pulsed drive. Explosion damages are found to differ significantly between n-Si and p-Si substrate cases and are explained by different carrier-multiplications processes: in the n-Si case, impact ionization propagates from SiO_2_ to Si, causing highly-localized melt explosions of Si in the depletion region, whereas in the p-Si case, from SiO_2_ towards graphene electrode, resulting in laterally wide-spread micro-explosions. These findings are expected to help optimally design the GOS-based atomizer structure and its operation for low voltage, small-volume analyte, high sensitivity chip-scale emission spectroscopy. For example, the different lateral damage-extents (<~300 μm diameter for n-Si GOS and >1 mm diameter for p-Si GOS case) set the limits of spatial resolution of probing and optimum analyte amount/coverage for maximum sensitivity. Micro-plasma systems have been reported in literature for miniature chemical analysis of liquid or gas phase analytes^39–41^. Compared to those microfabricated microfluidic/microchamber devices, the GOS-based atomizer offers unique advantages: low voltage operation (<~50 V), small volume analytes (in solid or liquid phases), and simplicity of sample preparation for chip-scale emission spectroscopy.

## Electronic supplementary material

supplementary info
