# Supplementary material for: Electrically-triggered micro-explosion in a graphene/SiO2/Si structure
Source: Sci Rep. 2018 May 9;8:7379. doi: 10.1038/s41598-018-25776-z (PMC5943326; doi:10.1038/s41598-018-25776-z)
Supplement: Supplementary file 1 — supplementary info [file 41598_2018_25776_MOESM1_ESM.pdf]

Supplementary Information

Electrically-triggered micro-explosion in a graphene/SiO_2/Si structure

Siyang Liu, Myungji Kim, and Hong Koo Kim

1. Calculation of electric field distributions in GOS structure

The gate voltage (V_g) applied to a GOS structure can be expressed as a summation of flat band voltage (V_{FB}), surface potential (φ_s) and oxide voltage drop (V_{ox}):

$$V_g = V_{FB} + \varphi_s + V_{ox}$$

where flat band voltage (V_{FB}) is equal to graphene work function (ϕ_{gr}) minus silicon work function (ϕ_{Si}):

$$V_{FB} = \phi_{gr} - \phi_{Si}$$

The space charge density (Q_s) in silicon side can be obtained by solving the Poisson equation and is expressed as follows [S1]:

$$Q_s = -\varepsilon_0 \varepsilon_{Si} E_{Si} = \sqrt{\frac{2\varepsilon_0 \varepsilon_{Si} q p}{\beta}} \left[\left(e^{-\beta\varphi_s} + \beta\varphi_s - 1 \right) + \frac{n}{p} \left(e^{\beta\varphi_s} - \beta\varphi_s - 1 \right) \right]^{1/2}$$

$$\beta = \mathrm{q/kT}$$

The voltage drop across the oxide layer (V_{ox}) is related to the space charge (Q_s) and oxide capacitance ($C_{ox} = \varepsilon_{ox}/ d$) as follows.

$$V_{ox} = Q_s/C_{ox}$$

Graphene work function (ϕ_{gr}) is the sum of intrinsic graphene work function (ϕ_{gr_int} = 4.56 eV) and its Fermi level shift (ΔE) under electric field:

$$\phi_{gr} = \phi_{gr\_int} + \Delta E$$

For monolayer graphene (MLG), the Fermi level shift (ΔE_1) can be expressed as follows (positive for negative gate voltage, negative for positive gate voltage):

$$\Delta E_1 = \pm \hbar |\nu_F| \sqrt{\pi n_s}$$

which involves reduced Planck constant ($\hbar$ = 6.58×10^{-16} eV·s), Fermi velocity (ν_F = 1.1×10^{8} cm/s) and carrier concentration (n_s = Q_S/q: Q_S is space charge density and q is elementary charge).

For bilayer graphene (BLG), the Fermi level shift (ΔE_2) is expressed as follows (positive for negative gate voltage, negative for positive gate voltage) [S2]:

$$\Delta E_2 = \pm \hbar^2 \pi n_s / 2m^*$$

which involves effective mass of carrier in BLG ($m^* = 0.033 m_e$: m_e is the electron rest mass).

Solving the above equations simultaneously, the followings are calculated as a function of applied gate bias voltage V_g in the range of 0 to 50 V: surface potential (φ_s); surface charge density (Q_s); electric field strength at the interface of silicon and oxide (E_{Si}); and depletion width (W): See Figures 1 - 3 and Tables 1 & 2.

Figure 1 shows the graphene work functions (of both monolayer and bilayer graphene cases) plotted as a function of gate voltage applied to our graphene/SiO_2/Si (GOS) structure under reverse bias. Note that the amount of Fermi level shift remains negligible for bilayer graphene case: both p-Si and n-Si GOS structures under large bias voltage (up to 50 V). Based on this result, we assume the Fermi level of 8-layer-graphene GOS to be the same as the binary graphene case.

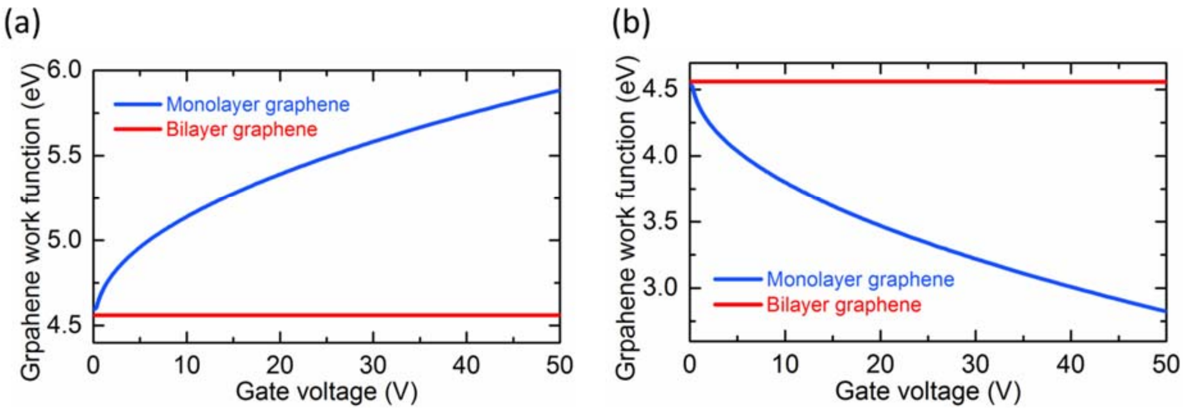

Supplementary Figure 1. Gate voltage versus graphene work function in graphene/SiO_2/Si structure with monolayer graphene and bilayer graphene under inversion bias on (a) p-Si substrate and (b) n-Si substrate.

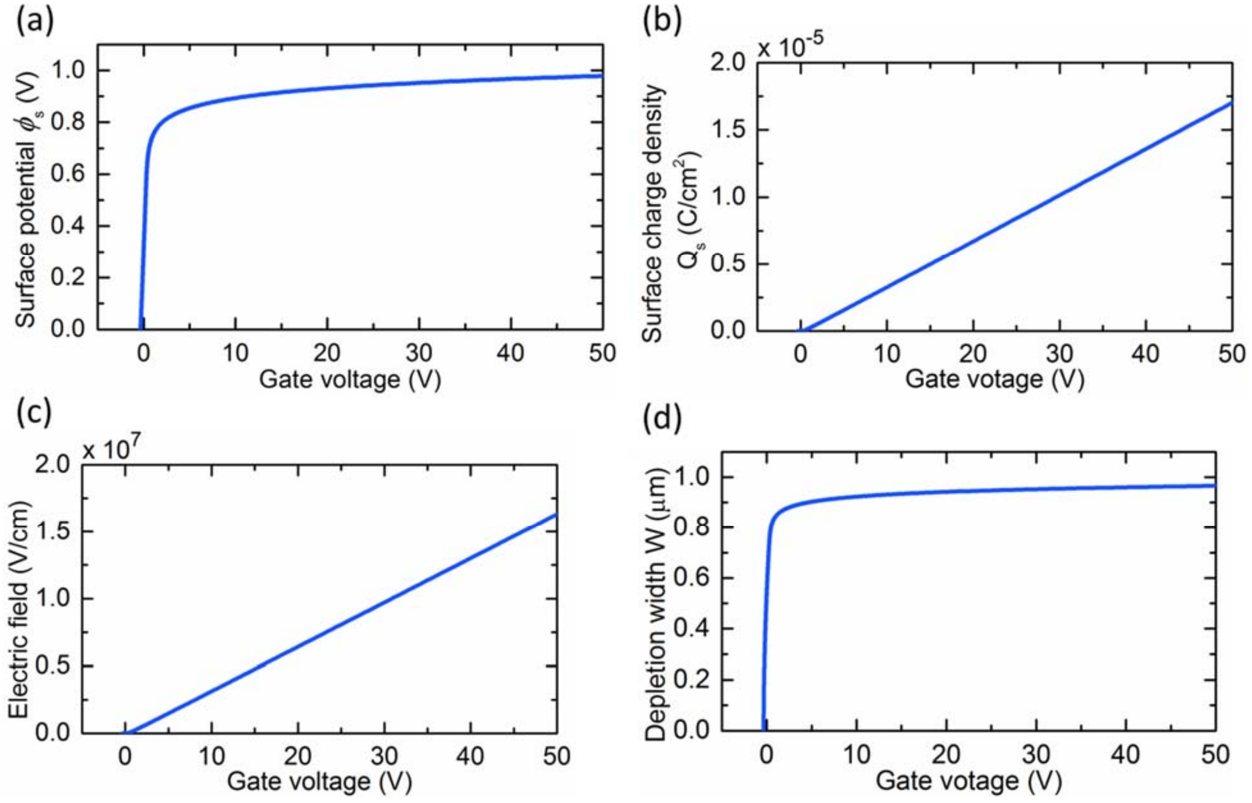

Supplementary Figure 2. Surface potential (a), surface charge density (b), electric field at the Si/SiO_2 interface (c) and depletion width (d) of 8LG/(10 nm)SiO_2/p-Si under reverse bias. Gate electrode is positively biased from -0.5 V to 50 V.

Hole concentration: N_A	$1.368 \times 10^{15} \mathrm{cm}^{-3}$		
Electron concentration: n	$1.537 \times 10^{6} \mathrm{cm}^{-3}$		
p-type silicon work function: ϕ_{pSi}	4.91 V		
Flat band voltage: V_{FB}	− 0.35 V		
Voltage	10 V	30 V	50 V
Surface potential: φ_s	0.89 V	0.95 V	0.98 V
Oxide voltage drop: V_{ox}	8.76 V	28.7 V	48.67 V
Oxide field: E_{ox}	8.76 MV/cm	28.7 MV/cm	48.67 MV/cm
Surface charge density: Q_s	$3.27 \times 10^{-6} \mathrm{C/cm^2}$	$1.02 \times 10^{-6} \mathrm{C/cm^2}$	$1.78 \times 10^{-5} \mathrm{C/cm^2}$

Electric field at Si/SiO_2 interface: E_{Si}	3.13 MV/cm	9.73 MV/cm	16.4 MV/cm
Depletion width: W	9.23×10^{-5}cm	9.52×10^{-5}cm	9.65×10^{-5}cm
Depletion charge density: Q_{dep}	$2.02 \times 10^{-8}C/cm^2$	$2.08 \times 10^{-8}C/cm^2$	$2.11 \times 10^{-8}C/cm^2$
Maximum depletion electric field: E_{dmax}	$1.92 \times 10^4 V/cm$	$1.98 \times 10^4 V/cm$	$2.00 \times 10^4 V/cm$

Supplementary Table 1. Summary of key parameter calculation for 8LG/10 nm SiO_2/p-Si.

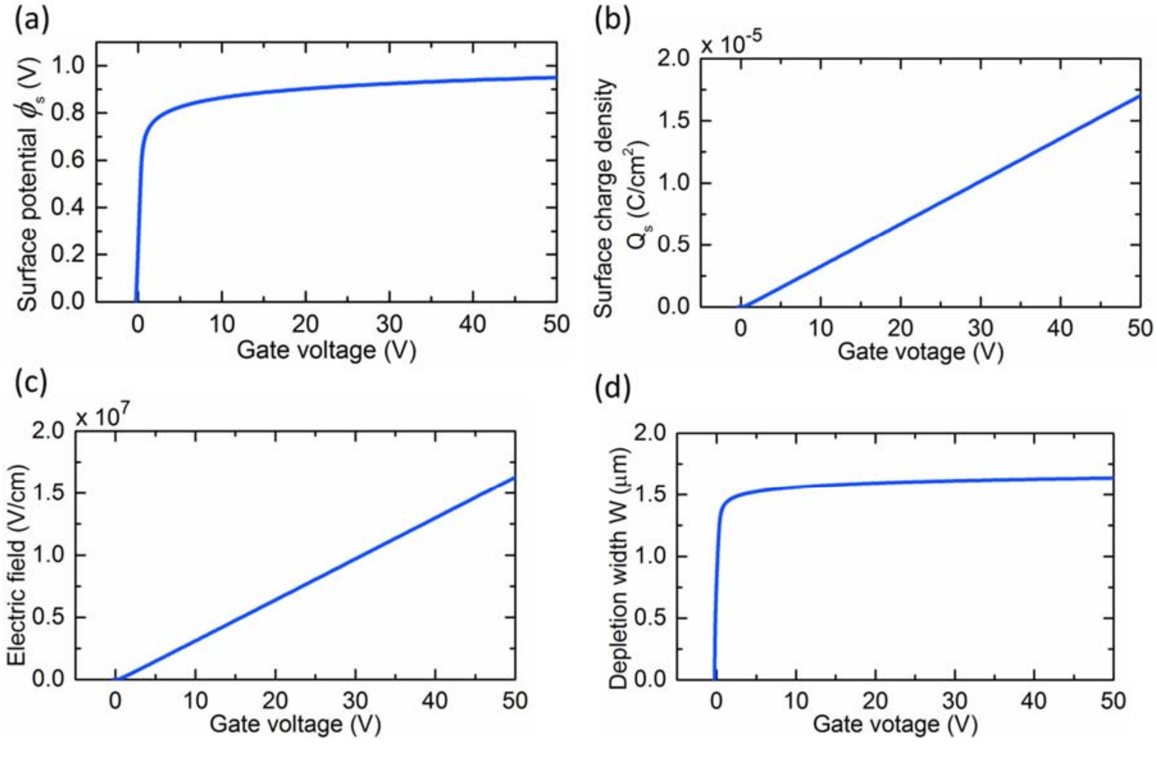

Supplementary Figure 3. Surface potential (a), surface charge density (b), electric field at the Si/SiO_2 interface (c) and depletion width (d) of 8LG/(10 nm)SiO_2/n-Si under reverse bias. Gate electrode is negatively biased from -0.5 V to 50 V.

Electron concentration: N_D	$4.623 \times 10^{14} \mathrm{cm}^{-3}$		
Hole concentration: p	$4.548 \times 10^{6} \mathrm{cm}^{-3}$		
n-type silicon work function: ϕ_{nSi}	4.31 V		
Flat band voltage: V_{FB}	0.25 V		
Voltage (reverse bias)	10 V	30 V	50 V
Surface potential: φ_s	0.86 V	0.93 V	0.95 V
Oxide voltage drop: V_{ox}	8.89 V	28.82 V	48.8 V
Oxide field: E_{ox}	8.89 MV/cm	28.82 MV/cm	48.8 MV/cm
Surface charge density: Q_s	$3.24 \times 10^{-6} \mathrm{C/cm^2}$	$1.01 \times 10^{-6} \mathrm{C/cm^2}$	$1.70 \times 10^{-5} \mathrm{C/cm^2}$
Electric field at Si/SiO_2 interface: E_{Si}	3.10 MV/cm	9.70 MV/cm	16.3 MV/cm
Depletion width: W	$1.56 \times 10^{-4} \mathrm{cm}$	$1.61 \times 10^{-4} \mathrm{cm}$	$1.64 \times 10^{-4} \mathrm{cm}$
Depletion charge density: Q_{dep}	$1.15 \times 10^{-8} \mathrm{C/cm^2}$	$1.19 \times 10^{-8} \mathrm{C/cm^2}$	$1.21 \times 10^{-8} \mathrm{C/cm^2}$
Maximum depletion electric field: E_{dmax}	$1.09 \times 10^{4} \mathrm{V/cm}$	$1.13 \times 10^{4} \mathrm{V/cm}$	$1.15 \times 10^{4} \mathrm{V/cm}$

Supplementary Table 2. Summary of key parameter calculation for 8LG/10 nm SiO_2/n-Si.

2. Calculation of thermal energy needed for vaporization of Si

The thermal energy required for heating, melting and vaporizing of Si is calculated using the formula, $\Delta Q = m(c_p \Delta T + \Delta_{fus} H + \Delta_{vap} H)$. Here m is the mass of local Si exploded, c_p is the specific heat, and $\Delta_{fus}H$ and $\Delta_{vap}H$ are the enthalpies of fusion and vaporization, respectively. ΔT is the temperature difference between room temperature (300 K) and the vaporization point. The following values were used for material constants of Si [S3]. Vaporization point: 3,538 K. $\Delta_{fus}H$: 1,787 J/g. $\Delta_{vap}H$: 1.6×10^4 J/g. Specific heat capacity, c_p: 0.71 J/g-K. Density: 2.33 g/cm^3. Volume of Si at micro-explosion site: π x $(10\mu m)^2$ x $(1\mu m) = 3.14 \times 10^{-10}$ cm^3. Si mass exploded: 7.32×10^{-10} g. The required thermal energy ΔQ is estimated to be 1.5×10^{-5} J.

Supplementary References

[S1] Sze, S. M. *Physics of Semiconductor Devices.* 2nd ed. (Wiley, 1981).

[S2] Ziegler, D. et al. Variations in the work function of doped single-and few-layer graphene assessed by Kelvin probe force microscopy and density functional. Phys. Rev. B 83, 235434 (2011).

[S3] Lide, D. R. ed. (2006) *CRC Handbook of Chemistry and Physics*, 87th ed. (CRC Press, New York), p4-134, 5-18, 6-114.
